# Neurogenic orthostatic hypotension in Parkinson’s disease: is there a role for locus coeruleus magnetic resonance imaging?

**DOI:** 10.1007/s00702-023-02721-7

**Published:** 2023-11-30

**Authors:** Giovanni Palermo, Alessandro Galgani, Gabriele Bellini, Francesco Lombardo, Nicola Martini, Riccardo Morganti, Davide Paoli, Sara De Cori, Francesca Frijia, Gabriele Siciliano, Roberto Ceravolo, Filippo Sean Giorgi

**Affiliations:** 1 Center for Neurodegenerative diseases–Parkinson’s disease and Movement disorders, Unit of Neurology, Department of Clinical and Experimental Medicine, University of Pisa, Pisa, Italy; 2 Department of Translational Research and of New Surgical and Medical Technologies, University of Pisa, Pisa, Italy; 3 Department of Radiology, Fondazione Monasterio/CNR, Pisa, Italy; 4 Deep Health Unit, Fondazione Monasterio/CNR, Pisa, Italy; 5 Section of Statistics, University of Pisa, Pisa, Italy; 6 Bioengineering Unit, Fondazione Monasterio/CNR, Pisa, Italy

**Keywords:** Dysautonomia, Locus coeruleus, Magnetic resonance imaging, Orthostatic hypotension, Parkinson’s disease

## Abstract

Locus coeruleus (LC) is the main noradrenergic nucleus of the brain, and degenerates early in Parkinson’s disease (PD). The objective of this study is to test whether degeneration of the LC is associated with orthostatic hypotension (OH) in PD. A total of 22 cognitively intact PD patients and 52 age-matched healthy volunteers underwent 3 T magnetic resonance (MRI) with neuromelanin-sensitive T1-weighted sequences (LC-MRI). For each subject, a template space-based LC-MRI was used to calculate LC signal intensity (LC contrast ratio—LC_CR_) and the estimated number of voxels (LC_VOX_) belonging to LC. Then, we compared the LC-MRI parameters in PD patients with OH (PD^OH+^) versus without OH (PD^OH−^) (matched for sex, age, and disease duration) using one-way analysis of variance followed by multiple comparison tests. We also tested for correlations between subject’s LC-MRI features and orthostatic drop in systolic blood pressure (SBP). PD^OH−^ and PD^OH+^ did not differ significantly (*p* > 0.05) based on demographics and clinical characteristics, except for blood pressure measurements and SCOPA-AUT cardiovascular domain (*p* < 0.05). LC_CR_ and LC_VOX_ measures were significantly lower in PD compared to HC, while no differences were observed between PD^OH−^ and PD^OH+^. Additionally, no correlation was found between the LC-MRI parameters and the orthostatic drop in SBP or the clinical severity of autonomic symptoms (*p* > 0.05). Conversely, RBD symptom severity negatively correlated with several LC-MRI parameters. Our results failed to indicate a link between the LC-MRI features and the presence of OH in PD but confirmed a marked alteration of LC signal in PD patients.

## Introduction

The locus coeruleus (LC) is the main noradrenergic (NA) nucleus of the brain, giving rise to diffuse projections throughout the whole central nervous system (CNS) (Poe et al. 2020).

Extensive neuropathological evidence in humans indicates that there is a marked LC neuronal loss in Parkinson’s disease (PD) and that a significant LC degeneration occurs since the early stages of disease, years before the onset of motor symptoms (Iranzo et al. 2014; Oertel et al. 2019).

However, LC degeneration estimation in vivo has been obtained only recently by magnetic resonance imaging (MRI) with LC-sensitive sequences (LC-MRI) (Sasaki et al. 2006).

LC dysfunction is associated with several non-motor symptoms of PD, such as anxiety, depression, REM behavior disorder (RBD), cognitive disturbances, apathy, and fatigue (Remy et al. 2005; Tredici and Braak 2013).

Orthostatic hypotension (OH) is a common and debilitating non-motor manifestation of PD with a point prevalence of 30% (Velseboer et al. 2011). The presence of OH in early disease stages is associated with a poor prognosis, clustering with cognitive deficits and RBD in a malignant phenotype of PD (Fereshtehnejad et al. 2015). Importantly, RBD and impaired cognitive functioning have been both related to LC dysfunction as underlying mechanism (Paredes-Rodriguez et al. 2020). Thus, it is not surprising that, although the causes of OH in PD are multifactorial, there is increasing evidence that NA dysfunction plays a prominent role. In particular, although OH is classically thought to be mainly the result of a NA cardiac denervation and peripheral NA deficiency, accompanied by impaired baroreflexes (Jain and Goldstein 2012), a damage to the LC complex has been also strongly related to its occurrence (Sommerauer et al. 2018).

MRI has been successfully used to study LC integrity, but only very recently a more standardized methodological framework for LC imaging analysis has been suggested (Betts et al. 2019; Giorgi et al. 2022; Galgani et al. 2023).

In this study, we hypothesize that the central NA system is more severely affected in PD patients with OH (PD^OH+^) compared to PD patients without OH (PD^OH−^), and thus, we analyzed LC through MRI in these two groups of subjects.

## Materials and methods

Twenty-two patients with PD were specifically enrolled for this study at the Center for Parkinson’s Disease and Movement Disorders of the Unit of Neurology of the University of Pisa, while 53 age- and sex-matched cognitively intact and neurologically healthy controls (HC) were part of a previously published study (Galgani et al. 2023). The LC-MRI protocol was approved by the Ethical Committee of Pisa University Hospital, which was conducted in accordance with Helsinki Declaration, and written informed consent was obtained before LC-MRI (prot.#1203, PE-2013–02349574). The diagnosis of PD was made according to the MDS clinical diagnostic criteria for Parkinson’s disease (Postuma et al. 2015). Other inclusion criteria were as follows: Hoehn and Yahr scales between 1 and 3 and stable dosage of dopaminergic medications for at least 4 weeks before the evaluation. We excluded patients with dementia, according to current consensus clinical diagnostic criteria (Emre et al. 2007).

Firstly, we included patients with OH (PD^OH+^), defined as a fall in systolic blood pressure (SBP) ≥ 20 mmHg or diastolic BP (DBP) ≥ 10 mmHg within 3 min of standing. Measurement of supine and standing heart rate (HR) was used to confirm that patients had neurogenic OH (nOH) (ΔHR/ΔSBP ratio lower than 0.5 bpm/mmHg after 3 min in the standing position) (Norcliffe-Kaufmann et al. 2018; Guaraldi et al. 2020). Baseline blood pressure was defined as the mean of two measurements on the upper right arm with the participant in the supine position after 5 min of rest. The measurements were repeated in a standing position after 1, 3, and 5 min. We adopted stringent exclusion criteria to account for as many potentially existing confounding variables as possible for OH, excluding eventually candidates (among both patients and HC) with diabetes, hypertension, and other disorders potentially associated with autonomic dysfunction, severe intracranial or extracranial artery stenosis/occlusion, clinical history of acute cerebrovascular disease, history of peripheral arterial disease, or taking medications for heart or other drugs such as tricyclic antidepressants and alpha-adrenergic antagonists (e.g., for prostate disorders) that influence orthostatic challenge. Other general exclusion criteria were severe medical comorbidities, psychiatric illnesses, and MRI signs of moderate–severe chronic vascular encephalopathy, according to Fazekas et al. (1987).

Then, for every PD patient with nOH, we looked for PD subjects without OH (PD^OH^) but with similar age (± 2 years) and disease duration (± 2 years) and matched by gender with the abovementioned PD^OH+^ subjects.

Each patient underwent full neurological examination at the time of BP assessment. Motor symptoms were assessed with the Unified Parkinson’s Disease Rating Scale (UPDRS III). The MOntreal Cognitive Assessment (MoCA), SCales for Outcomes in Parkinson’s Disease (SCOPA), Hamilton Anxiety Rating Scale (HAM-A), Hamilton Depression Rating Scale (HDRS), and REM Sleep Behavior Disorder Screening Questionnaire (RBDSQ) were also collected.

### MRI protocol

LC imaging was performed using a 3-Tesla MRI unit (GE Excite HDx, GE, USA) with an 8-channel phased-array head coil. A 2D-FSE T1-weighted sequence was registered with the following parameters: TR, 600 ms; TE, 14 ms; flip angle, 90°; echo train length, 2; NEX, 5; matrix size, 512 × 384; FOV, 200 × 200 mm; pixel size, 0.39 × 0.52 mm; contiguous slices, 12, slice thickness, 2.2 mm, slice gap, 0; and acquisition time, 14.29 min). The images were acquired at the level of the anatomical location of LC (Fernandes et al. 2012), covering an area from the inferior border of the pons to the posterior commissure, along the oblique axial plane and perpendicular to the fourth ventricle floor.

The post-acquisition analysis was performed profiting from a study-specific template, whose detailed description is reported in (Giorgi et al. 2022) and (Galgani et al. 2023). The scans of patients were warped from the native space to the template space, and LC mask was applied. We calculated the LC contrast ratio “LC_CR_” (i.e., the ratio between the intensity detected within the LC mask and the one of the reference pontine regions), as an index of LC signal intensity, and “LC_VOX_” as an estimate of LC voxels belonging to LC. For each subject, we calculated the LC_CR_ and LC_VOX_ of the entire LC both for left (“left LC”) and right (“right LC”) hemispheres.

### Statistical analysis

Demographics and clinical variables were compared between PD^OH+^ and PD^OH−^ using an independent *t*-test (*p* < 0.05). The data were first tested for normality with the Kolmogorov–Smirnov test. Because most MRI data exhibited normal distributions, parametric statistics were applied. Between-group differences in LC_CR_ and LC_VOX_ measures were tested using parametric one-way ANOVA followed by post hoc analysis. Partial Pearson correlation coefficient analysis was used to assess associations between LC_CR_/LC_VOX_ parameters and blood pressure values as well as between LC_CR_/LC_VOX_ and the other motor and non-motor symptoms (depression, anxiety, RBD, cognition, and other dysautonomic symptoms than OH) evaluated at the time of BP assessment. Statistical analysis was conducted using the Statistical Package for the Social Sciences (SPSS), version 26.

## Results

### Demographics and clinical assessment

Demographics and baseline clinical characteristics of the population included in this study are reported in Table 1. Twenty-two PD subjects were recruited. However, one PD^OH−^ patient was excluded from the final analysis due to MRI movement artifacts. PD groups were matched for age, sex, and disease duration. Among all PD participants, the mean (SD) age was 71.4 (5.4) years and mean (SD) disease duration was 6.2 (3.4) years. There were no differences in LEDD, and disease severity between PD^OH+^ and PD^OH−^ and both groups had similar scores in non-motor scales (MoCA, SCOPA-AUT total score, HAM-A, HDRS, and RBDSQ). As expected, PD^OH+^ had significant SBP and DBP than PD^OH+^ and worse scores in the cardiovascular domain of the SCOPA-AUT scale (Table 1).

**Table 1:** Demographic and clinical features of study population

	PD^OH+^ (*n* = 11)	PD^OH−^ (*n* = 11)	Controls *(n* = 52)	p-value*
Age (yrs)	71.18 ± 5.67	71.10 ± 5.36	71.71 ± 7.8	0.97
Female/male	2/9	2/9	20/32	
MoCA	24.11 ± 3.72	24.73 ± 2.61	28.11 ± 1.8	0.669
Age at onset (yrs)	65.18 ± 5.25	64.73 ± 4.67		0.832
Disease duration (yrs)	6.00 ± 3.55	6.36 ± 3.36		0.807
LEDD (mg)	433.27 ± 187.64	560 ± 369.99		0.323
Clinostatic SBP (mmHg)	154.27 ± 20.90	139 ± 16.31		0.07
Clinostatic DBP (mmHg)	93.45 ± 11.38	81.27 ± 10.04		**0.015**
Clinostatic HR (bpm)	75.27 ± 15.54	77.36 ± 8.31		0.698
Orthostatic SBP 1′ (mmHg)	122.91 ± 22.58	127.91 ± 15.42		0.551
Orthostatic DBP 1′ (mmHg)	80.45 ± 12.94	81.91 ± 10.90		0.779
HR 1′	81.18 ± 15.46	83.82 ± 10.32		0.643
Orthostatic BP 3′ (mmHg)	120.09 ± 19.01	130.18 ± 16.88		0.203
Orthostatic DBP 3′ (mmHg)	77.91 ± 12.43	78.27 ± 9.98		0.94
HR 3′ (bpm)	79.91 ± 13.74	82.55 ± 11.78		0.634
Orthostatic SBP 5′ (mmHg)	121.56 ± 23.64	128.09 ± 18.71		0.498
Orthostatic DBP 5′ (mmHg)	76.22 ± 17.41	80.73 ± 11.22		0.492
HR 5′ (bpm)	87.67 ± 21.05	82.73 ± 9.78		0.53
Systolic drop 3′ (mmHg)	34.18 ± 15.87	8.82 ± 7.92		**0.0001**
Diastolic drop 3′ (mmHg)	15.55 ± 9.36	3 ± 1.47		**0.001**
MDS-UPDRS III	22.4 ± 11.18	25.18 ± 7.53		0.508
SCOPA-AUT total score	19.44 ± 6.29	15.91 ± 6.94		0.253
Gastrointestinal subscore	5.11 ± 3.52	5.09 ± 3.11		0.989
Urinary subscore	6.22 ± 3.83	5.45 ± 3.48		0.644
Cardiovascular subscore	3.44 ± 1.74	0.82 ± 1.47		0.002
Thermoregulatory subscore	1.89 ± 1.27	2.55 ± 3.05		0.527
Pupillary subscore	0.22 ± 0.67	0 ± 0.00		0.347
Sexual subscore	2.78 ± 1.39	2.09 ± 1.58		0.322
HDRS	8.78 ± 4.84	9.73 ± 6.60		0.724
HAM-A	13 ± 6.67	14.64 ± 10.27		0.686
RBDSQ	6 ± 2.87	4.18 ± 3.46		0.224

Data are expressed as mean ± standard deviation

*PD*^OH−^: Parkinson’s disease patients without orthostatic hypotension; *PD*^OH+^: Parkinson’s disease patient with orthostatic hypotension; *LEDD*: levodopa equivalent dose; *SBP*: systolic blood pressure; *DBP*: diastolic blood pressure; *HR*: heart rate; *SCOPA-AUT*: Scales for Outcomes in Parkinson's Disease Autonomic Questionnaire; *MDS-UPDRS*: Movement Disorder Society-Sponsored Revision of the Unified Parkinson's Disease Rating Scale; *MoCA*: Montreal cognitive assessment scale. *HDRS*: Hamilton Depression Rating Scale; *HAM-A*: Hamilton Anxiety Rating Scale; *RBDSQ*: REM Behavior Disorder Screening Questionnaire

**When comparing PD^OH+^ and PD^OH−^ groups

Results in bold characters indicate significant difference *p* < 0.05

### LC group differences

**Fig. 1:**
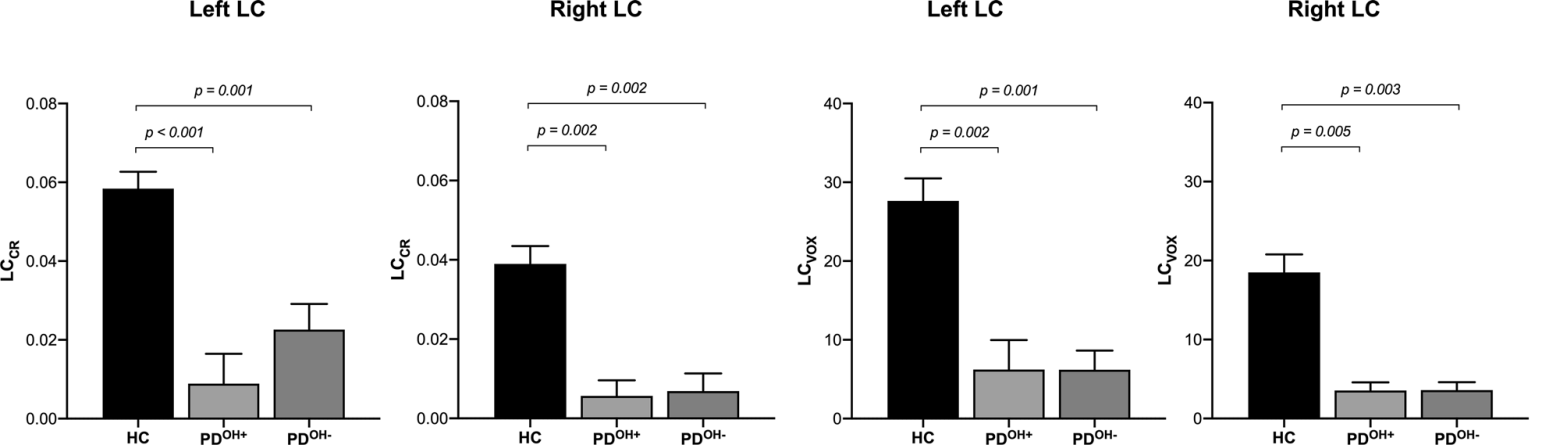
Differences of locus coeruleus (LC)–magnetic resonance imaging (MRI) features among diagnostic groups

**Fig. 2:**
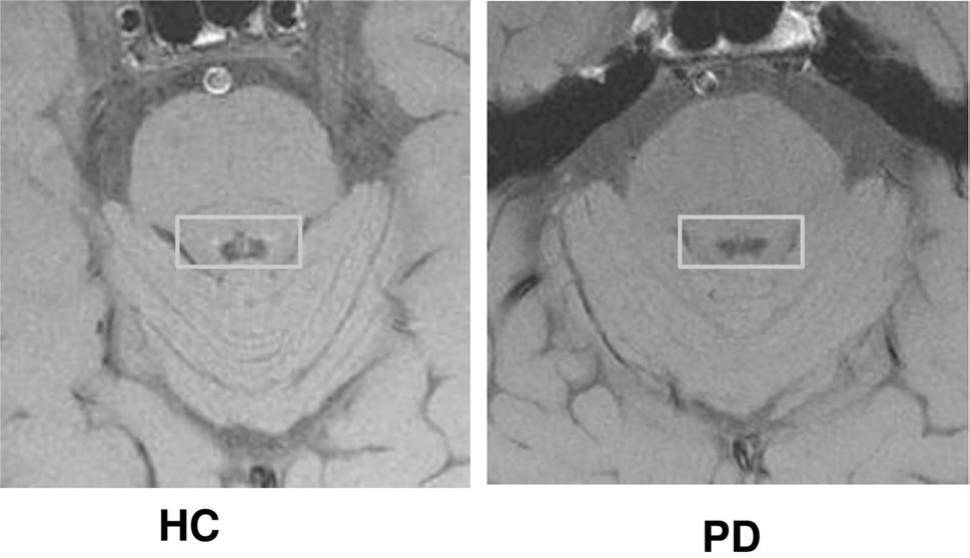
Representative locus coeruleus (LC)–magnetic resonance imaging (LC-MRI) images from PD and healthy controls (HC). The figure shows representative images from 2D-FSE T1-weighted sequence in native space (before post-acquisition LC data extraction, see methods) in corresponding slices at the level of the pons, from one PD and one HC. In each slice, a box insert shows the region where the two LC are placed (visualized just laterally to the median plane, below the floor of the fourth ventricle). In PD, there is a marked decrease in LC-related intensity compared with HC

ANOVA analysis revealed a statistically significant difference in LC_CR_ and LC_VOX_ among the three groups both for the right LC and the left LC (Fig. 1). In particular, both PD groups had significantly lower LC_CR_ and LC_VOX_ than HC group (a representative image of two subjects is reported in Fig. 2), but there was no significant difference in LC parameters between PD^OH+^ and PD^OH−^ (Fig. 1). We obtained the same results when performed comparison on LC_CR_ and LC_VOX_ for each (rostral and caudal) subregion of the LC (data not shown).

### Relation between LC parameters and clinical variables

**Fig. 3:**
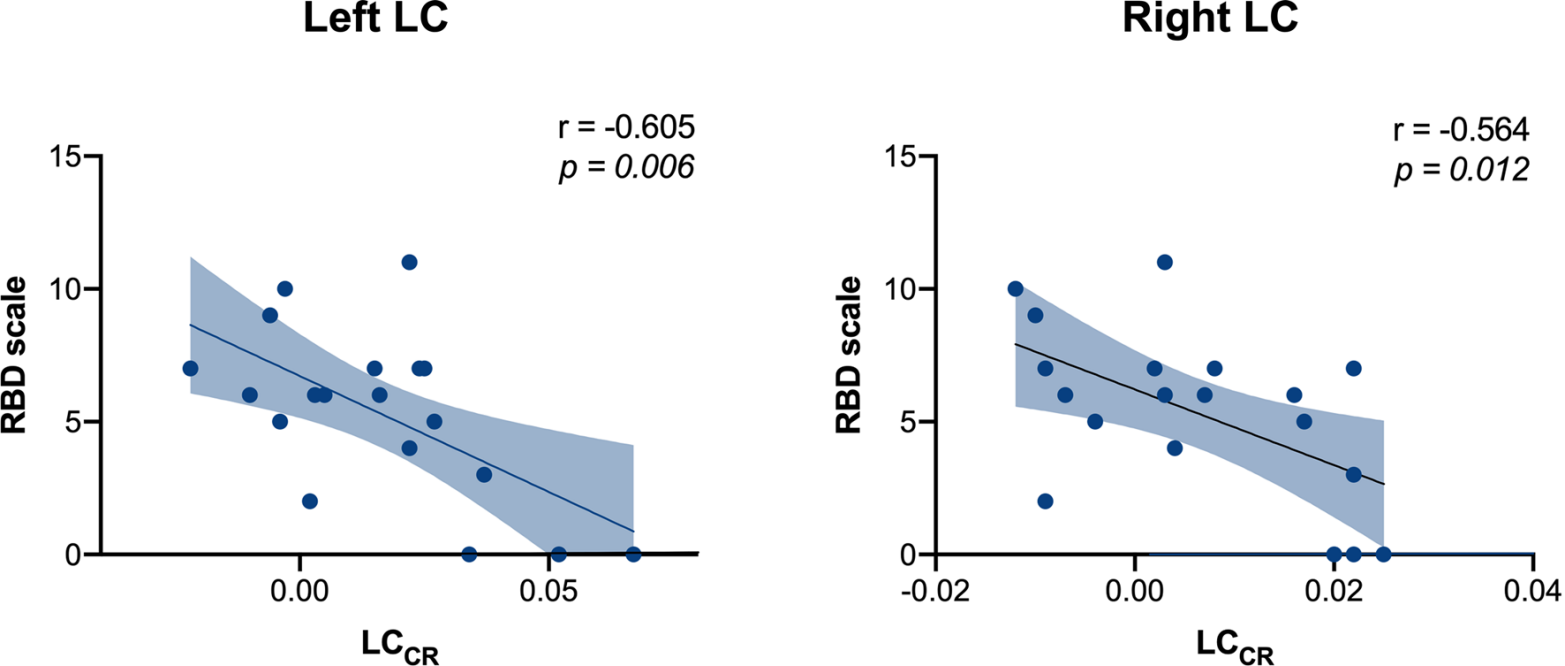
Correlation between LC contrast ratio [LC_CR_] of the left and right LC with the REM sleep behavior disorder screening questionnaire (RBDSQ) score

Linear correlation analysis in the included PD subjects did not show any relevant correlation between the single subject’s LC-MRI parameters and neither the drop in blood pressure nor the SCOPA-AUT total score and domain scores. Conversely, RBD symptom severity negatively correlated with LC-MRI parameters (Fig. 3).

## Discussion

In this study, we explored the under-investigated link between degeneration of LC and OH in PD through MRI, and our data suggest that LC degeneration is not strictly associated with nOH. Our findings indicate that LC is significantly altered in all PD patients when compared with healthy individuals, but no differences in the PD population were found with regard to the presence of OH. Conversely, in line with our predictions, we found evidence for a link between RBD score and LC degeneration.

The exact pathophysiology of OH in PD is incompletely understood. Patients with PD tend to demonstrate predominantly peripheral autonomic failure (Coon 2020). Defective NA release from postganglionic sympathetic neurons and impaired compensatory baroreflex response are thought to be the primary pathophysiological mechanisms of nOH in PD (Jain and Goldstein 2012). Moreover, deposition of alpha-synuclein (α-syn) along the central autonomic network may also contribute to nOH (Coon et al. 2018). The nucleus of the solitary tract and rostral ventrolateral medulla have been shown to play an important role in the activation of the baroreflex system, receiving the modulation, among others, of the LC itself through both direct and indirect connections (Bockstaele et al. 1989; Sun 1995; Giorgi et al. 2021; Samuels and Szabadi 2008). Although the LC is among the first brain regions to degenerate in PD (Braak et al. 2003), few studies examined the structural degeneration of LC in the pathogenesis of PD non-motor symptoms (Malatt and Tagliati 2022), likely reflecting previous technical difficulties in visualizing in vivo the LC because of its small size and physiological inter-subject variability (Fernandes et al. 2012). However, in the last decade, LC-MRI data have been shown to be able to provide a quite reliable surrogate for LC integrity determined histopathologically (Keren et al. 2015), after the improvement of specific MRI sequences, and the development of standardized analysis protocols. Specifically, the combination of neuromelanin with ions and macromolecules within LC neurons likely contributes to a T1-shortening effect in fast spin-echo sequences and to T1 prolongation at magnetization transfer-weighted imaging (Galgani et al. 2020). In PD, a significant reduction of LC-MRI intensity has been confirmed (Castellanos et al. 2015; Schwarz et al. 2017), even greater than that the *substantia nigra* MRI one (Isaias et al. 2016).

Our results confirmed both a significant reduction of LC-MRI signal intensity in PD patients versus controls and the lack of correlation between MRI-based LC parameters and disease duration or motor severity (Ohtsuka et al. 2014; Prasuhn et al. 2021; Doppler et al. 2021). Nevertheless, LC analysis may be theoretically useful for identifying patients suffering from non-motor symptoms. It shows an association between altered LC signal and RBD (García-Lorenzo et al. 2013; Knudsen et al. 2018), depression (Solopchuk et al. 2018; Wang et al. 2018, Ye et al. 2022; Madelung et al. 2022), and cognitive dysfunction (Prasuhn et al. 2021; Li et al. 2019). Interestingly, regionally specific pattern of LC changes in PD, with the middle-caudal portion being more affected than the rostral part, has been associated with cognitive impairment and apathy (Ye et al. 2022; Madelung et al. 2022). A regional association with the left caudal LC has been also found for OH in 42 PD patients (of whom 17 had OH) in a recent study investigating the potential link between orthostatic BP drop and LC-MRI signal changes (Madelung et al. 2022). We failed to find an association between OH and LC-MRI parameters in the present study, which was specifically designed to test the hypothesis of a different LC-MRI signal in PD patients with and without nOH, matched for age, sex and disease duration. The same held true when we tested for regional (rostral and caudal) differences in neurodegeneration within the LC, in line with postmortem studies reporting a uniform neuronal loss over the entire LC in PD (German et al. 1992). Besides, we did not see an overall significant association between LC integrity and the severity of other non-motor symptoms (depression, anxiety, and cognition), except for RBD. This finding is not unexpected since LC-MRI signal intensity has been shown to be significantly decreased even in RBD patients without PD (Ehrminger et al. 2016). Interestingly, in the present study, the relationship between RBD and LC was confirmed both for the caudal and rostral portion of LC (not shown).

RBD often co-occurs with poor cognition and OH, together contributing to define a rapidly progressive subtype of PD whose biological underpinning could result from NA deficiency (Espay et al. 2014). Sommerauer and colleagues (2018) tested this hypothesis in a combined ^11^C-MeNER (a radioligand specific for NA transporter) PET and LC-MRI study in PD patients with and without RBD and healthy controls. They detected that there is an association between the NA system as measured with ^11^C-MeNER PET and all three groups and that PD patients with RBD displayed the lowest neuromelanin signal in LC; however, LC-MRI signal did not significantly correlate neither with RBD, nor with cognitive performance or with BP changes (Sommerauer et al. 2018). Similarly, we did not find a relationship between LC degeneration and orthostatic drop in BP, and LC-MRI signal was significantly lower in all PD patients versus controls, without differences between patients with and without OH, in line with a recent neuropathological study in forty-four patients with PD/Lewy body disease which failed to indicate an association of LC pathology with the presence of OH (Tong and Chen 2021). One potential explanation for these negative imaging and pathological findings might be the inclusion in the above-reported studies of subjects with intermediate/advanced disease stage, as LC degeneration in PD starts early, and thus, LC-MRI may potentially disclose differences among those PD subgroups at the early stage only or even in the prodromal phases of the disease. In fact, LC could already be significantly damaged when motor symptoms firstly appear (Braak et al. 2003) This may account for the failure of LC-MRI to distinguish between early and advanced PD (Ohtsuka et al. 2014) and to identify, as in our study, differences between PD subgroups in a cohort evaluated after a mean disease duration of follow-up of 6 years.

In contrast, NA terminal integrity, measured through ^11^C-MeNER PET, has been linked to the expression of OH and other clinical PD symptoms more consistently than LC nuclear integrity assessed with LC-MRI (Sommerauer et al. 2018; Kelberman et al. 2020). A disease process beginning in the distal axon and proceeding retrogradely (similarly to what was proposed for the dopaminergic nigrostriatal pathway degeneration (Cheng et al. 2010)) could explain, at least in part, the discrepancy between structural and functional imaging modalities in studying the NA neurons of the LC itself. However, adaptive circuit changes, the heterogeneity among LC projection neurons, and their large axonal arborization are other factors potentially contributing to this discrepancy. In addition, it appears that LC-MRI and PET findings are not closely correlated even in healthy subjects, suggesting that the two compartments of the NA system (cell bodies and axon terminals) could be independent of each other (Helmich and Lehéricy 2021).

In contrast to our study, none of the previous ones distinguished between nOH and non-nOH. Moreover, we included only PD^OH+^ who had never taken any drugs affecting autonomic function and without comorbidities potentially contributing to autonomic disturbances. However, several limitations, such as the small sample size of PD patients and the single-center nature of the study, should be acknowledged. The relatively small number of participants with PD did not allow performing additional analyses, but these numbers are aligned with those of the only two studies published thus far on the relationship between LC-MRI signal intensity and OH (Sommerauer et al. 2018; Madelung et al. 2022). Besides, we acknowledge that the cardiovascular autonomic assessment was not comprehensive since it was limited to the study of BP and heart rate, while RBD was measured only through the RBD questionnaire and not by polysomnography. Furthermore, although there was no significant difference in LEDD between PD^OH+^ and PD^OH−^, the impact of anti-PD medication on OH cannot be ruled out. Finally, it is worth mentioning that in our study the HC group had been included exclusively for comparison of LC-MRI with PD groups, but, since BP parameters had not been systematically assessed in these HC subjects, we could not perform an association analysis of LC-MRI and BP parameters along the whole spectrum of LC degeneration; however, this did not affect the main purpose of the analysis which was based on the comparison between PD^OH+^ and PD^OH−^.

In conclusion, we did not find any difference in LC-MRI parameters in PD patients with and without neurogenic OH, while we confirmed a significant LC degeneration in all PD patients compared with controls, suggesting that changes in LC signal might not be useful for identifying PD patients with cardiovascular dysautonomia, at least at this stage of the disease. Future studies with larger and early/prodromal PD cohorts with longitudinal follow-up will provide greater insights about the potential link between the integrity of LC and OH.

## Data Availability

The data that support the findings of this study are available from the corresponding author upon reasonable request.
